# Congenital peritoneal encapsulation—a rare entity presented with small bowel obstruction

**DOI:** 10.1093/jscr/rjaa601

**Published:** 2021-01-30

**Authors:** André Tojal, Júlio Marques, Sandra Coelho, Maria-João Ferreira, Noel Carrilho, António Horta-Oliveira, Carlos Casimiro

**Affiliations:** Department of General Surgery, Centro Hospitalar Tondela-Viseu, E.P.E., Av. Rei D. Duarte, Viseu, Portugal; Department of General Surgery, Centro Hospitalar Tondela-Viseu, E.P.E., Av. Rei D. Duarte, Viseu, Portugal; Department of General Surgery, Centro Hospitalar Tondela-Viseu, E.P.E., Av. Rei D. Duarte, Viseu, Portugal; Department of General Surgery, Centro Hospitalar Tondela-Viseu, E.P.E., Av. Rei D. Duarte, Viseu, Portugal; Department of General Surgery, Centro Hospitalar Tondela-Viseu, E.P.E., Av. Rei D. Duarte, Viseu, Portugal; Department of General Surgery, Centro Hospitalar Tondela-Viseu, E.P.E., Av. Rei D. Duarte, Viseu, Portugal; Department of General Surgery, Centro Hospitalar Tondela-Viseu, E.P.E., Av. Rei D. Duarte, Viseu, Portugal

**Keywords:** peritoneal encapsulation, intestinal obstruction, intestinal malrotation

## Abstract

Congenital peritoneal encapsulation is a rare congenital malformation in which all or part of the small bowel is covered by a thin accessory peritoneal membrane. Despite being usually asymptomatic and an incidental finding during surgery or autopsy, there is a small number of reports in the literature whose diagnosis was established in the context of intestinal obstruction. The authors review the topic and describe a case report undergoing surgery for intestinal obstruction. Intraoperatively, there was a partial peritoneal encapsulation of the small bowel with signs of intestinal malrotation. Peritoneal membrane excision, terminal ileum release and complementary appendicectomy were performed. There was a favorable clinical evolution in the postoperative period. Although rare, it is important to remember this entity in the differential diagnosis of patients with intestinal obstruction, in the absence of other etiologic factors.

## INTRODUCTION

Congenital peritoneal encapsulation (CPE) is a rare, congenital entity in which the small bowel is surrounded by an accessory peritoneal membrane. Its diagnosis is usually accidental, during surgery or autopsy [1]. Abdominal cocoon syndrome (ACS) and sclerosing encapsulating peritonitis (SEP) are also conditions associated with encapsulation of the small bowel, the first being idiopathic and the second associated with chronic peritoneal dialysis [2, 3]. Although there are some cases described in the international literature with presentation of intestinal obstruction, this is extremely rare [1].

## CASE REPORT

A 41-year-old male patient, without past medical or surgical history, denying communicable disease exposure or previous trauma history, presented to the emergency department with colicky epigastric abdominal pain associated with bilious vomiting, but normal bowel transit the day before. The patient was at the hospital the previous day with similar complaints. The physical examination was normal and he was discharged after analgesia, being asymptomatic.

**Figure 1:**
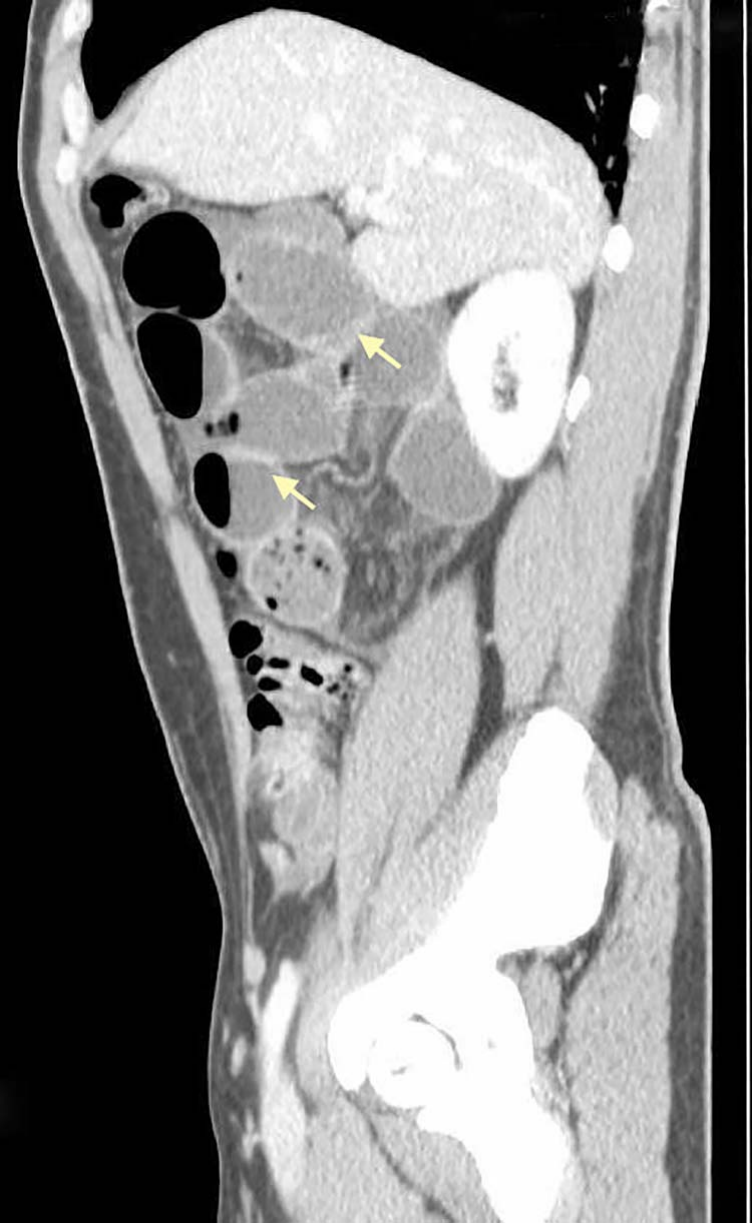
Sagittal CT scan shows small bowel distension with air/fluid levels (arrows).

**Figure 2:**
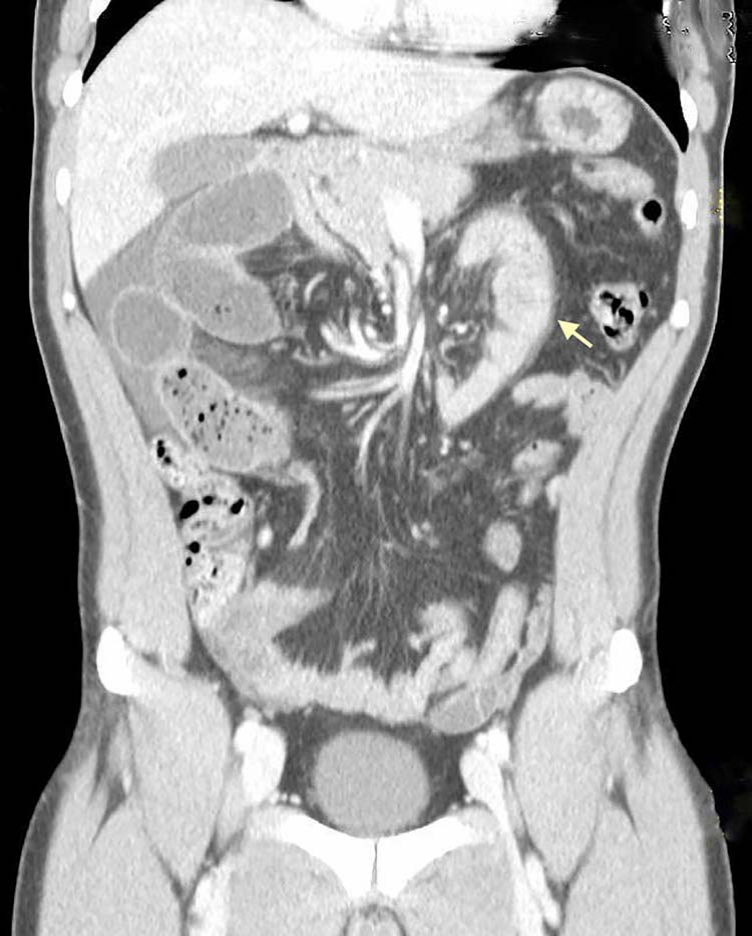
Coronal CT scan shows signs of intestinal malrotation, with alteration of the normal topography of the duodenal arch (arrow).

**Figure 3:**
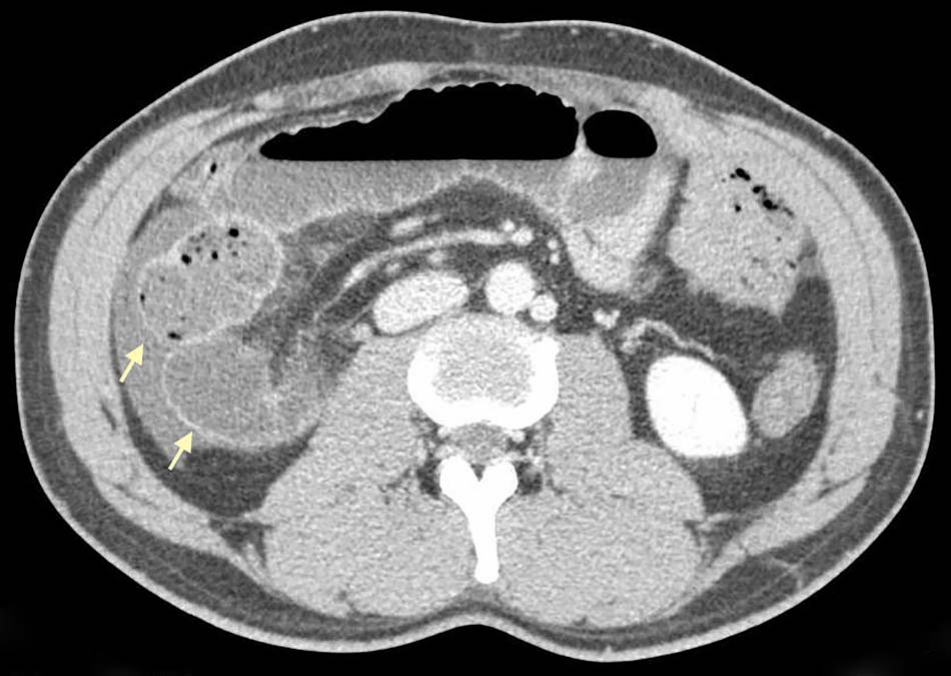
Axial CT scan shows retrocecal position of terminal ileum and free fluid in the right abdominal quadrants (arrows).

On physical examination, he was eupneic, hemodynamically stable and apyretic. His abdomen was soft, not distended, painless, with normal bowel sounds. Rectal examination showed an empty ampulla. Blood analysis revealed mild leukocytosis and little increase in C-reactive protein. Plain radiograph of the abdomen revealed dilated small bowel loops with air/fluid levels. Abdominal ultrasound revealed swollen intestinal loops on the right flank and interloop fluid. For further clarification, abdominal and pelvic computed tomography (CT) scan showed small bowel distension, air/fluid levels and free fluid (Fig. 1); signs of intestinal malrotation, with alteration of the normal topography of the duodenal arch (Fig. 2) and retrocecal position of terminal ileum (Fig. 3).

**Figure 4:**
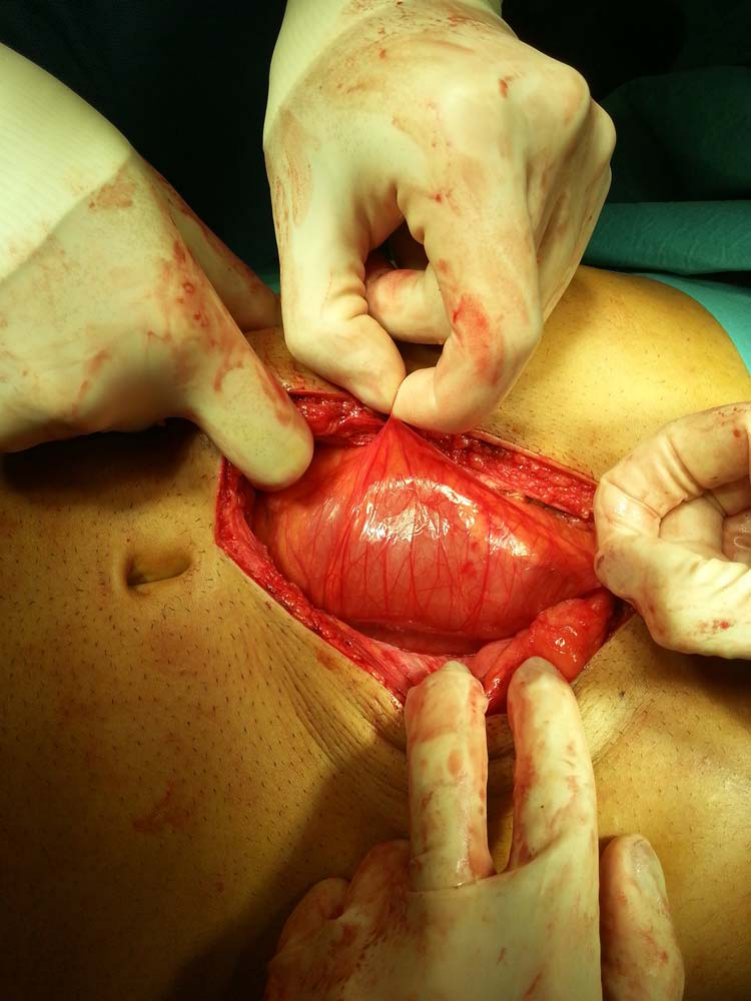
Peritoneal encapsulation of the small bowel at laparotomy.

**Figure 5:**
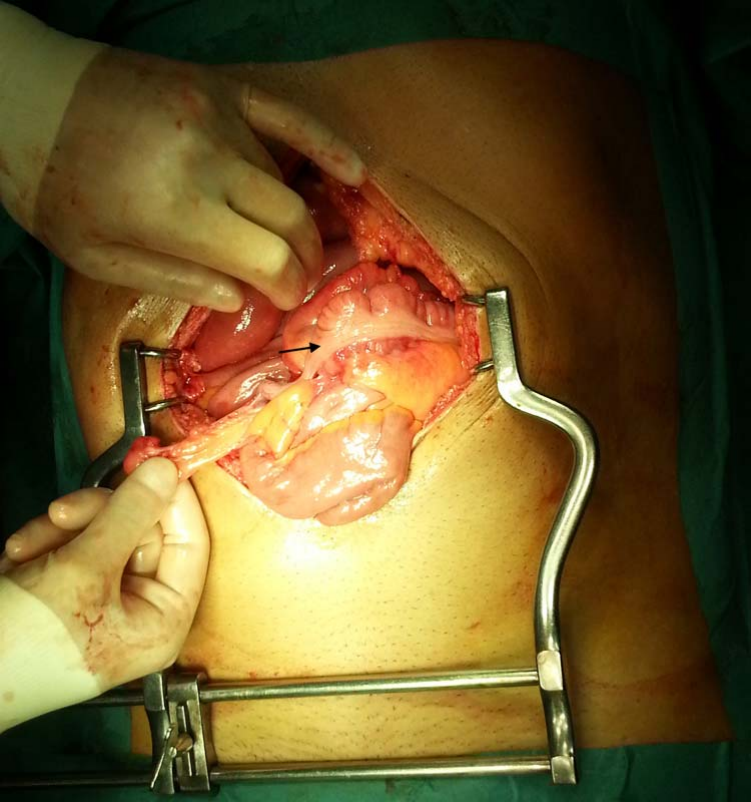
After excision of the accessory peritoneal membrane, signs of intestinal malrotation with medialization of the right colon were noted (arrow).

**Figure 6:**
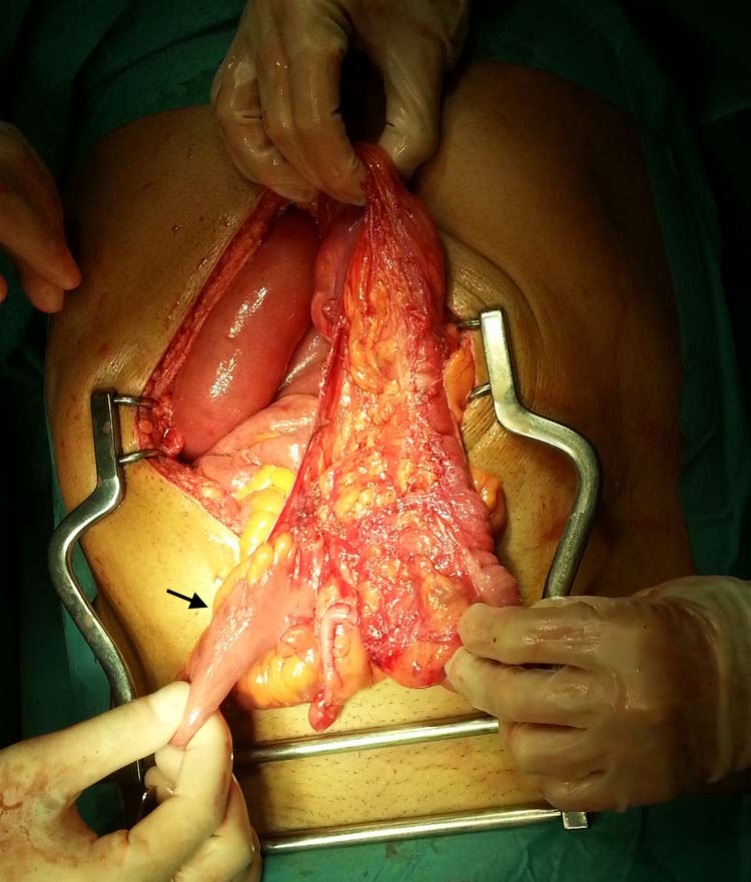
Terminal ileum in a retrocecal position leading to intestinal obstruction (arrow).

In the presence of an intestinal obstruction of an unclear cause, an exploratory laparotomy was proposed. A peritoneal encapsulation (Fig. 4) of the small bowel was identified, without intestinal ischemia. The peritoneal sac was located in the right abdominal quadrants, only partially involving the small bowel, so in the left quadrants there was normal caliber ileum externally to it. The membrane was excised and the peritoneal cavity was explored, showing signs of intestinal malrotation with medialization of the right colon (Fig. 5) and a retrocecal position of the terminal ileum leading to intestinal obstruction (Fig. 6). After lysis of adhesions from the bowel to the membrane, the terminal ileum was released and complementary appendectomy was done. Histopathology revealed normal peritoneal tissue. After prolonged ileus, patient was discharged on the 8th postoperative day and presented asymptomatic 1 year later.

## DISCUSSION

CPE is a rare congenital malformation in which the small bowel is partially or totally involved by an accessory peritoneal membrane derived from the peritoneum of the yolk sac [1, 4]. It develops during the 12fth week of embryological development when there is an abnormal return of the physiological umbilical hernia, containing the midgut, to the peritoneal cavity [5]. It was first described by Cleland in 1868 [6], with fewer than 50 cases described in the literature [1, 7], the diagnosis of most cases being accidental. It occurs more frequently in young male patients and may be associated with intestinal malrotation [1, 4]. The fixation points of this peritoneal sac are laterally the ascending and descending colon, the transverse colon superiorly and the posterior parietal peritoneum inferiorly [6]. While mostly asymptomatic, there is reference to repeated episodes of abdominal pain or intestinal obstruction [1–3]. In cases associated with small bowel obstruction, two signs suggestive of the disease have been described [7, 8]: (i) asymmetric and permanent distention of the abdomen, which does not vary with peristaltic activity, and (ii) different consistency of the abdominal wall on palpation.

Although preoperative diagnosis is difficult, imaging findings may suggest the diagnosis. CT may reveal aggregation of small bowel loops involved by the peritoneal membrane and signs of intestinal obstruction [1, 4]. A helical pattern of the distended small bowel has been described—helix sign [1, 2].

Surgical treatment involves excision of the peritoneal membrane and lysis of adhesions from its fixation points, since the bowel loops within the capsule lie freely without adherence to each other [1, 2, 5, 7]. Histological examination of the membrane reveals normal peritoneum with fibrovascular tissue covered by mesothelium, without inflammation [1, 4, 7]. Intestinal resection is reserved for situations in which an atraumatic excision of the membrane is not possible or in the presence of intestinal ischemia. The recognition and better understanding of this entity has currently allowed a laparoscopic approach [1, 7]. The concomitant performance of appendectomy was due to the presence of intestinal malrotation, to avoid future diagnostic doubts in the face of abdominal pain.

SEP was first described by Owtschinnkow in 1907 [1–3, 7], initially termed ‘peritonitis chronica fibrosa incapsulata’. This is an acquired condition associated with chronic peritoneal dialysis, recurrent peritonitis, ventriculoperitoneal/peritoneovenous shunts, sarcoidosis, tuberculosis, Mediterranean fever, protein-S-deficiency, liver transplantation, systemic lupus erythematosus and fibrogenic foreign bodies [1, 4].

ACS was first described by Foo *et al* [9] in 1978. It typically occurs in female adolescents from tropical/subtropical countries, although there are cases described in temperate climates [1, 4, 9, 10]. It is usually considered idiopathic; however, there are several theories, namely tubal reflux during menstruation with viral overinfection, retrograde peritonitis, immune-mediated tissue destruction triggered by gynecological infection [1, 3, 4]—probably the result of subclinical peritonitis. In these cases, there is an opaque fibrous membrane not covered by mesothelial cells that may involve small bowel, colon or solid organs [1]. Unlike CPE, it usually presents with an acute abdomen requiring exploratory laparotomy [1]. Yip and Lee [10] recognized four clinical features suggestive of the diagnosis: (i) a young female patient without an obvious cause of intestinal obstruction, (ii) past similar episodes that resolved spontaneously, (iii) presenting with abdominal pain and vomiting but rarely abdominal distension and (iv) the presence of a non-tender soft mass on abdominal palpation. Its treatment also involves excision of the membrane [1, 3].

The actual incidence of CPE is difficult to determine, given the difficulty in differential diagnosis with ACS and SEP, entities in which the small bowel is also involved by an accessory peritoneal membrane. Contrasting from SEP, whose postoperative mortality can reach 60–80% [1, 4], CPE has an excellent prognosis after surgery, being associated with high percentage of survival and no reported cases of recurrence [1, 4, 5], with longest follow-up being 7 years of symptom-free survival [1]. Therefore, in this case, an annual clinical evaluation was proposed with imaging/endoscopic evaluation according to symptoms, because the risk of peritoneal adhesions formation after any surgical procedures in the peritoneal cavity and the presence of intestinal malrotation that can lead to bowel obstruction.

CPE is a rare clinical entity to bare in mind in the differential diagnosis of intestinal obstruction in which other causes have been excluded. A high index of suspicion allows for timely surgical intervention, avoiding ischemia and consequent intestinal resection, which are associated with greater morbidity and mortality.
